# Occupational future time perspective and mental health problems across adolescence: Random‐intercept cross‐lagged panel analysis and alternative variations

**DOI:** 10.1002/jad.12438

**Published:** 2024-10-22

**Authors:** Yi Yang, Ingrid Obsuth, Xinxin Zhu, Denis Ribeaud, Manuel Eisner, Aja Murray

**Affiliations:** ^1^ Department of Psychology University of Edinburgh Edinburgh UK; ^2^ Clinical and Health Psychology University of Edinburgh Edinburgh UK; ^3^ Jacobs Center for Productive Youth Development University of Zurich Zürich Switzerland; ^4^ Institute of Criminology University of Cambridge Cambridge UK

**Keywords:** ADHD, externalizing, future time perspective, internalizing, occupational future time perspective, random intercept cross‐lagged panel model

## Abstract

**Introduction:**

Adolescence is a crucial developmental stage characterized by escalating mental health issues as well as an increasing awareness of future career possibilities. Occupational future time perspective has been shown to be a promotive factor for social functioning and mental health, and a component in evidence‐based clinical practices and randomized controlled trial intervention studies. However, it requires more rigorous and ecological corroboration from longitudinal analysis at the within‐person level.

**Methods:**

Random intercept cross‐lagged panel models with several adjustments and sensitivity analyses were applied to the longitudinal data from the Zurich Project on the Social Development from Childhood to Adulthood (Zurich, Switzerland), to analyze how occupational future time perspective and psychological/neurodevelopmental outcomes (attention deficit hyperactivity disorder symptoms/externalizing/internalizing problems) covaried across ages 13 (*N* = 1365), 15 (*N* = 1446), and 17 (*N* = 1305) in the years 2016, 2018, and 2020, after controlling for sex (52% male), SES, and school type.

**Results:**

A small effect was found in a random intercept cross‐lagged panel model whereby occupational future time perspective at age 15 predicted externalizing problems at age 17 (*β* = .146, *p* = .05, [95% CI = 0.000, 0.292]), and in a random intercept (contemporaneous) reciprocal panel model specification attention deficit hyperactivity disorder symptoms at age 17 were contemporaneously associated with occupational future time perspective at age 17 (*β* = −.310, *p* < .05, [95% CI = −0.580, −0.041]). No cross‐lagged associations were found to be robust across different model specifications/adjustments.

**Conclusions:**

These findings suggest that improving occupational future time perspective may have limited impact on enhancing mental health, offering valuable insights for school‐based interventions. Further research and replication are necessary to confirm these results.

Adolescence, defined by the World Health Organization (WHO) as the second decade of life (Dick & Ferguson, 2015), is characterized by a burgeoning awareness of potential future career paths amidst a peak period for the onset and escalation of mental health issues (Solmi et al., 2022). Given this, there is considerable interest in identifying factors that relate to risk and resilience during this critical period, particularly in light of the evidence suggesting that resilience cultivated during adolescence can endure into adulthood (Werner & Smith, 2001).

Future time perspective (FTP) has emerged as such a promising resilience factor (Roepke & Seligman, 2016), particularly as it undergoes growth with increasing age during this critical developmental stage (Steinberg et al., 2009). Despite being conceptualized inconsistently across studies, FTP is inclusively defined as “a general concern for and corresponding consideration of one's future” as proposed by a systematic review and meta‐analysis that examined terminology employed across existing studies (Kooij et al., 2018). FTP involves diverse domains of life events. Research on FTP thus frequently subdivides this construct into domain‐specific sub‐concepts aligned with oriented content, such as educational (Andre et al., 2018), occupational (Henry et al., 2017), physical health (Murphy & Dockray, 2018), and environments domains (Milfont et al., 2012). Importantly, FTP has revealed negative associations with a spectrum of mental health and related issues, such as ADHD (Weissenberger et al., 2020; *r* = −0.16, *p* < .001, age range = 18–65), sensation seeking (Duangpatra et al., 2009; *r* = −0.25, *p* < .01, age range = 18–29), aggression (Stolarski et al., 2016; *r* = −0.20, *p* < .001, age range = 18–67), depression (*ρ* = −.34, *p* < .001), anxiety (*ρ* = −.23, *p* < .01), substance use (*ρ* = −.22, *p* < .001) and risk behavior (*ρ* = −.22, *p* < .001; mean age =32.5, k = 167; meta‐analysis by Kooij et al., 2018).

FTP arguably functions as a promotive factor (i.e., beneficial for all individuals, at both high and low levels of risk) compared to a protective factor (i.e., buffering particularly the negative effects from high levels of risk) (Dvorsky & Langberg, 2016; Masten, 2001; Wright & Masten, 2014; Wright et al., 2012). Its motivational potency across diverse life domains has received widespread recognition in the past decades (Andre et al., 2018; Henry et al., 2017; Kooij et al., 2018; Rudolph et al., 2018), prompting researchers to examine its underlying mechanism in promoting mental health. Cunningham et al. (2015) proposed a dual‐pathway framework (i.e., top‐down and bottom‐up) to explain the relationship between life circumstances, time perspective and mental health, indicating FTP appear to operate powerfully both directly in top‐down and indirectly in bottom‐up pathways. Another theory holds that the promotive function of FTP likely stems from its self‐regulation capacity towards future goal orientation. Baird et al.'s (2021) meta‐analysis on 2000 tests from 378 studies suggests that FTP operates through motivated self‐regulation processes, encompassing processes such as goal monitoring and goal pursuit, thereby facilitating favorable outcomes across life domains, including mental health outcomes such as aggression, substance use, and risk behaviors. It is also posited that the activation of regulatory focus, involving the selection and reordering of goal priorities, may serve as a developmental mechanism linking FTP to desirable life outcomes (Baltes et al., 2014).

Corroborating these theories, occupation FTP (OFTP), as a critical domain in FTP assessments, presents clear future goal orientations, as shown in assessments such as the Future Exceptional Scale developed by Wyman et al. (1993), and the questionnaires used by Peetsma et al. (2005), and by Burger and Mortimer (2021). Importantly, adolescents dedicate a significant portion of their time to educational endeavors, aiming to prepare for occupational or higher education and eventual employment. Thus, orientation toward future education and occupation increasingly emerges as an influential domain of expectation as they progress through secondary school. It is also noting that school experiences have been identified as significant contributors to the development of FTP in general (Shubert et al., 2020).

Additionally, OFTP has been shown to link to many mental health outcomes, such as reduced emotional exhaustion (*r* = −0.17, *N* = 3684, K = 4; see meta‐analysis by Rudolph et al., 2018), reduced marijuana use (Dennhardt et al., 2015), moderating the impact of problem‐focused strategies on psychological distress (Ho & Yeung, 2016), and mediating the relationship between job insecurity with depression, anxiety, and stress (Lam et al., 2019). Particularly, early positive FTP including occupational future have been shown to predict enhanced socioemotional adjustment among children (Wyman et al., 1993). Adolescents with strong OFTP in another study tended to experience fewer mental health issues, including conduct/externalizing, ADHD, depression, and anxiety, at a 1‐year follow‐up (Almroth et al., 2018). OFTP has also been shown to moderate the relationship between violence exposure and externalizing problems (So et al., 2018), and mediate the relationship between socioeconomic status and mental health outcomes, such as depression and anxiety (Gjerustad & Von Soest, 2012) among adolescents. Therefore, investigating how OFTP covary with adolescents’ mental health during adolescence holds paramount importance.

Aligned with these empirical findings, several randomized controlled trial (RCT) intervention studies also adopted OFTP as an intervention component. For instance, the Building Resiliency and Vocational Excellence (BRAVE) Program, designed to reduce substance use and violence, incorporates supplementary elements focusing on the development and monitoring of career goals, mentoring, peer‐to‐peer goal monitoring and reinforcement, vocational field trips, and more (Griffin et al., 2009). Similarly, the Lions–Quest Skills for Adolescence (SFA) curriculum includes components centered on setting positive future goals within its personal development unit, along with planning for the future in unit 7 (Eisen et al., 2003; Maalouf et al., 2019; Matischek‐Jauk et al., 2018). Notably, FTP and goal‐oriented strategies have also been integrated into evidence‐based intervention approaches, such as cognitive‐behavioral therapy (e.g., reducing catastrophising future, planning, goal setting; Roepke & Seligman, 2016) and acceptance and commitment therapy (e.g., goals as the actionable components of “value” element; Hayes et al., 2006). In summary, these academic and clinical practices underscore the potential efficacy of future‐focused interventions in mental health.

However, those clinical practices, despite allowing causal inferences, often encompass multiple components, rendering it challenging to disentangle their individual contributions. Particularly, the unique explanatory power of OFTP in relation to mental health needs clarification due to its salient role in educational practice during adolescence life. Further, while experiments can offer stronger support for causality, evidence from experimental manipulations (e.g., Rung & Madden, 2018) also necessitates ecological verification to ensure its applicability to real‐life scenarios, particularly concerning long‐term effects (e.g., over a 2‐year interval). For these reasons, longitudinal analysis of observational data can help provide important complementary evidence and ecological validation with the temporal associations.

To the best of our knowledge, none of previous related longitudinal studies (e.g., Fieulaine & Martinez, 2012; Jung et al., 2021; Konowalczyk et al., 2018; Lukavská, 2018) have elucidated the association at the intra‐individual level to establish stricter temporal predictions, such as distinguishing between‐person and within‐person effects to explore potential bidirectional temporal within‐person associations while considering between‐person confounds. The differentiation of between‐ and within‐person associations is facilitated by models such as the random intercept cross‐lagged panel model. Here the directional associations in individual deviations from the individual's own average level over time are examined. This includes assessing the possibility that deteriorations in mental health may impede adolescents’ capacity to anticipate and plan for their occupational future, while conversely, improved future occupational anticipations may alleviate mental health symptoms.

## 1 THE PRESENT STUDY

The relationships between OFTP and mental health outcomes at the within‐person level were the focus of the present study. The selection of internalizing, externalizing, and symptoms as mental health outcomes is informed by findings from transdiagnostic psychopathology research. Converging empirical evidence highlights two primary dimensions crucial for characterizing childhood and adolescent psychopathology: internalizing symptoms (e.g., anxiety, depression) and externalizing symptoms (e.g., aggression, delinquency, impulsivity) (e.g., Caspi et al., 2014; 2024). These symptoms also serve as targets for adolescent interventions incorporating FTP components (e.g., Griffin et al., 2009; Roberts et al., 2011) since they are among the most commonly observed problems among adolescents (Sonuga‐Barke et al., 2016). Previous evidence has suggested a link between OFTP and internalizing/externalizing symptoms (e.g., Rudolph et al., 2018; Skorikov & Vondracek, 2007). For example, social anxiety in adolescence is related to lower OFTP, which may have consequences for future educational pathways and later work life (Fernandez et al., 2023; Jystad et al., 2021). Higher levels of externalizing problems are associated with lower OFTP in adolescents (Metsäpelto et al., 2017). Additionally, ADHD stands out for examination for its prominent association with time‐processing abnormalities (e.g., Hart et al., 2012; Mette, 2023). ADHD may thus be likely impact the acquisition of FTP in the first place (Weissenberger et al., 2016). However, whether the acquisition of OFTP is linked to fewer ADHD symptoms remains uncertain.

Several additional factors, such as sex, socioeconomic status, and school type (high school vs. vocational education), merit consideration as potential confounding factors. Previous reviews and meta‐analyses have indicated that girls tend to score higher on FTP measures compared to boys (e.g., Andre et al., 2018) and sex/gender differences in specific mental health problems are well‐documented (e.g., Card et al., 2008; Faheem et al., 2022; Oldehinkel & Bouma, 2011). Moreover, favorable socioeconomic conditions have been linked to both a more positive FTP (Burger & Mortimer, 2021; Carvalho, 2015; Kooij et al., 2018), and better mental health (e.g., Flouri et al., 2014; McKay & Cole, 2017). Another important consideration is the heightened concern about future occupation during periods of key examinations and educational transitions, which can serve as pivotal moments in one's occupational trajectory. In Switzerland, for example, approximately 70% of adolescents undergo their final compulsory school examinations at age 15, coinciding with the application for vocational education and training programs (VET) (Bolli et al., 2021; Bosset et al., 2022). In contrast, adolescents in the upper echelons of the education system do not face such examinations until age 18. Consequently, adolescents on these different educational trajectories may exhibit variations in the immediacy of their concerns regarding future occupation.

Given the aforementioned considerations, particularly the focus on examining whether cross‐lagged effects still exist within a longitudinal framework after accounting for between‐person effects and controlling for stable confounding factors, we employed the Random Intercept Cross‐Lagged Panel Model (RI‐CLPM, Hamaker et al., 2015) with appropriate covariate adjustments to analyze longitudinal data spanning ages 13–17. Importantly, the awareness of future occupational expectations of students was increasing across ages 13, 15, and 17 due to the aforementioned rules that determine their future occupational paths (e.g., academic performance at age 15). Thus, the use of RI‐CLPM is further justified for the present study (Hamaker, 2023). Considering the existing significant evidence, the primary hypotheses of this study were as follows:

We hypothesized that there would be negative reciprocal developmental associations between OFTP and ADHD symptoms, externalizing problems, and internalizing problems, respectively, between ages 13, 15, and 17, after controlling for stable, between‐person differences in OFTP and mental health at baseline, as well as demographic variables including sex, socioeconomic status (SES), and school type.

## 2 METHODS

### 2.1 Participants

The present study acquired data from an existing longitudinal cohort and intervention study. The participants were from the Zurich Project on the Social Development from Childhood to Adulthood (z‐proso; Ribeaud et al., 2022). The z‐proso initially recruited a sample of 1675 children from 56 public primary schools in Zurich Switzerland, which were selected using a stratified random sampling procedure with considering school location and size. Schools were originally stratified based on school size (small or large) from seven school districts. The cohort data were collected repeatedly since the first time point in 2004 when participants aged 7 and the study remains ongoing to date. The present study used data from waves 5, 6, and 7 (ages 13, 15, and 17). A total of 1305 adolescents (with 90% born in Switzerland) participated at least once to fill out paper‐and‐pencil surveys in classrooms (or out of school time at later ages) when aging 13 (*N* = 1365), 15 (*N* = 1446), and 17 (*N* = 1305). The attrition rate was 2.6% from wave 5 to wave 6 and 11% from wave 6 to wave 7. Notably, attrition was higher among certain immigrant background groups, with the highest elevated odds of drop‐out observed in Albanian (OR = 3.24, *p* < .001), Portuguese (OR = 3.04, *p* < .001), and Serbian‐Croatian (OR = 3.45, *p* < .001) groups (Eisner et al., 2019). Approximately half of the sample was male (biological sex, male ≈ 52%), with the whole sample diverse in the background (e.g., 50.1% Switzerland, 6.4% Serbia‐Montenegro, 5.7% Sri Lanka, 5% Portugal, 4.1% Germany, 3.4% Turkey and 3% Italy primary caregiver nationality). It includes a slight overrepresentation of low socioeconomic status school districts. The missingness patterns were analyzed by Eisner et al. (2019) who found that nonresponse was higher amongst certain socially disadvantaged immigrant minority groups. Further information can be found in the z‐proso cohort profile (Ribeaud et al., 2022) and on the study website http://www.jacobscenter.uzh.ch/en/research/zproso/aboutus.html.

### 2.2 Ethics

Consistent with the principles of the Declaration of Helsinki, ethical approval of the z‐proso study was acquired from the Ethics Committee at the Faculty of Arts and Social Sciences of the University of Zurich. Before the investigation, active informed consent was signed by the adolescents themselves from age 13. Additionally, guardians had the option to withdraw their children from the study until the children reached the age of 18.

### 2.3 Measures

**Covariates**: The family socioeconomic status (SES) covariates include family household income and the highest education background of caregivers. The family household income was reported during the first wave when participants were age 7. The highest educational background of primary caregivers (both parents included) was reported at wave 5 (aged 13). The covariate, the sex of participants, was based on administrative records retrieved at baseline. The covariate, school type, was operationalised by forming two categories, “occupational education” and “gymnasium/general education.”

**Occupational Future Time Perspective**: for the consideration of age‐appropriate measures of time perspective, three OFTP items linking school performance to future jobs were included in waves 5, 6, and 7 of z‐proso. Waves 5–7 covered ages 13–17 when young people are transitioning from secondary schools to high schools/apprenticeships (e.g., in the z‐proso study, between 15 and 17, many secondary school adolescents move into apprenticeships, while those attending general education do not). This period is more likely to be the stage at which young people may start considering their future career directions. This is the rationale for the z‐proso study examining their OFTP at these three waves, and therefore, our study is limited by data availability since the OFTP information was not available before then. As part of a school‐related scale in z‐proso data collection, following previous investigation practice on OFTP, the future orientation towards school items included “When I grow up I want to have an interesting job, and I'm doing everything now to work towards that goal.” “I try hard at school to have a good job later in life.” and “Doing well at school is important to me.” Adolescents were asked to evaluate on a 4‐point Likert scale from 1= “false” to 4= “true.” Cronbach's alpha for the OFTP scale indicates acceptable internal consistency across different waves, with α = .73 at wave 5, α = .75 at wave 6, and α = .72 at wave 7. These items have also been found to have satisfactory inter‐item correlation, a metric which has been argued to be more appropriate for assessing reliability for scales with a low number items, with *r* = 0.473 at wave 5, *r* = 0.524 at wave 6, and *r* = 0.494 at wave 7 (Defoe et al., 2021; van Gelder et al., 2018).

**Social Behavior Questionnaire (SBQ)**: three dimensions (32 items in total) from the SBQ were included, with four items capturing the symptoms of attention‐deficit/hyperactivity disorder during the past 12 months (ADHD, including restlessness, difficulties to concentrate, inattentive, hectic and fidgety, score range 4–20), nine internalizing items covering depression and anxiety during the past month (e.g., sad without reason, feel along, worried, fear; score range 9–45), 19 externalizing items covering oppositional defiant disorder, nonaggressive symptoms of conduct and aggression during the past 12 months (e.g., violent attack, activity exclusion, yell at parent, lie to parent, score range 19–95). The SBQ is designed to capture dimensional variance rather than act as a diagnostic tool, previous evidence suggests that it has favorable psychometric properties for this purpose for ADHD traits and internalizing and externalizing problems (Murray et al., 2019a, 2020). Importantly, it has also shown longitudinal invariance up to the metric level in adolescence (Murray et al., 2019b). All item responses were on a 5‐point Likert scale from 1= “Never” to 5= “Very Often.”

### 2.4 Statistical analysis

We conducted the following comprehensive considerations and model comparisons to ensure rigorous analyses and reliable results. Composite scores for ADHD symptoms, externalizing problems, and internalizing problems (mean score across related items for each subscale after reverse scoring the reverse‐worded items) were used for analysis in the models. Several approaches are possible for disaggregating between‐ and within‐person effects, such as (autoregressive) latent trajectory models with structured residuals (LTM‐SR, also referred to as the latent curve model with structured residuals, LCM‐SR in Curran et al., 2014), and random intercept cross‐lagged panel models (RI‐CLPMs), and the particular choice of which to adopt requires some consideration. According to a study comparing longitudinal models to examine reciprocal relations, if the key interest is in the reciprocal associations between developmental processes controlling for time‐stable covariates, the RI‐CLPM is arguably the better choice (Usami et al., 2019). RI‐CLPM is a flexible option because it neither assumes invariance of effects over time nor imposes a particular form of developmental change (although it is restrictive in the sense of assuming all participants follow the same developmental trajectory as encoded in the grand means). Further, from a pragmatic standpoint, one study compared 7 cross‐lagged models including LTM‐SR and found that the RI‐CLPM stably converged in every sample, while other models frequently showed convergence problems (Orth et al., 2021). We, therefore, adopted the RI‐CLPM approach to examine how OFTP and mental health problems (ADHD symptoms, externalizing problems, and internalizing problems, respectively) covaried over time (across ages 13, 15, and 17), reciprocally at the within‐person level in particular.

The RI‐CLPM, as an extension of the cross‐lagged panel model (CLPM, Hamaker et al., 2015), allows separating the observed variable scores into the group‐level associations (random intercept covariances) and individual‐level parameters (residuals aside from the random intercept) at each time point. RI‐CLPM provides a more nuanced and accurate analysis of longitudinal data than CLPM by separating and controlling for stable between‐person differences and focusing on within‐person changes over time. This leads to more precise and interpretable estimates of the dynamic processes that occur within individuals, making RI‐CLPM a suitable choice for the present study. Additionally, separating within‐person from between‐person variability and controlling for stable individual differences can help address shared method bias (e.g., social desirability, recall bias, and response style tendencies), as the data were all collected using self‐report questionnaires. After separating the stable random intercept correlations, the residuals at each time point are modeled in terms of autoregressive, cross‐lagged and within‐time/concurrent estimates at the within‐person level. The autoregressive parameters capture carry‐over effects, specifically, whether deviations from one's baseline level of a construct predict deviations at the next time point (e.g., whether being higher than usual on a construct at one time point predicts being higher than usual at the next). The cross‐lagged effects represent the extent to which the two variables at a given time point influence each other at the next time point after controlling for the prior level of the variables (i.e., adjusting for the autoregressive effects). After controlling for the autoregressive and cross‐lagged effects, (residual) covariances can be estimated to capture concurrent covariation of the two included variables at each time point. The residual covariances then capture the concurrent relationship of the residual of the within‐component of the first variable, and the residual of the within‐component of the second variable after accounting for lagged relations. We allowed covariances between these residuals. Under this specification, the effect of OFTP (or mental problems) at time point t‐1 on mental problems (or OFTP) at time point t is adjusted for time point t‐1 OFTP (or mental problems), which works for both cross‐lagged pathways.

Given the potential impact of sex and socioeconomic conditions on OFTP and mental health problems as shown in previous studies (Andre et al., 2018; Card et al., 2008; Faheem et al., 2022; Flouri et al., 2014; Kooij et al., 2018; McKay & Cole, 2017; Oldehinkel & Bouma, 2011), these covariates were adjusted for by regressing the random intercept factors on the covariates (sex, SES, school type). This adjusts the between‐person association for their effects. In this way, the covariates are treated as time‐invariant and may explain between‐person variation in time perspective and mental health. To test the assumption of stable effects of the covariates (against the possibility of time‐varying effects), an analysis was conducted for the covariates by comparing the Bayesian Information Criterion (BIC) values of the two types of models: models assuming time‐invariant covariates with stable effects by regressing the random intercept factors on all baseline covariates, and models assuming time‐varying effects of these covariates by regressing the variables directly on the covariates at each time points. The ΔBIC was calculated using the higher BIC value to minus the lower BIC value of the two comparing models, with ΔBIC > 10 as the standard to support the model with lower BIC as the best model.

The specific sample size for each of the RI‐CLPMs was slightly different due to the missing data pattern for each of the variables, provided in Table S2 in the supplementary materials. Models were fitted using robust maximum likelihood estimation (MLR) in MPlus 8.0 (Muthén & Muthén, 2017). Model fit standards for good fit from the RI‐CLPM were: CFI and TLI close to or above 0.90, and RMSEA and SRMR less than 0.08 (e.g., Hu & Bentler, 1999). Missing data were dealt with using the full information maximum likelihood (FIML) estimator inherent to MLR, which can provide unbiased estimates assuming missing at random (MAR). The use of a robust estimator was motivated by the fact that several of the variables analyzed showed non‐normal distributions.

In addition, it is also worth noting that RI‐CLPM models correctly identify long‐term reciprocal effects only under some conditions (Muthen & Asparouhov, 2023). In particular, neglecting concurrent associations in classic RI‐CLPMs sometimes can be problematic when variables measured at the same measurement occasion refer to different reference time‐frames (Speyer et al., 2023). Therefore, as suggested by Muthen and Asparouhov (2023) for RI‐CLPM analysis, additional models to verify whether the lagged (lag1, lag2) effects of RI‐CLPMs could rather be contemporaneous (lag0) were carried out. This consideration was based on two rationales. First, despite each wave of data for different variables being collected and labeled as the same wave, there were different reference periods for different variables in practice, which may bias to effect sizes of the RI‐CLPMs (for details see Muthen & Asparouhov, 2023; Speyer et al., 2023). The externalizing problems referred to behaviors during the past 12 months, ADHD referred to the past 12 months and internalizing problems to the past month, while the OFTP was worded in reference to the moment being interviewed. Thus, it was illogical theoretically for OFTP at the current moment to predict the mental problem behaviors in the past. A contemporaneous unidirectional rather than bidirectional effect was more reasonable in this situation. However, practically, at the within‐person level, there might be reporting biases because the data from the same wave were reported at the same time, despite the fact that different time frames were used in the survey. It is likely that the current OFTP state could bias the reporting of problems over the past year or month. Second, the true optimal time lag for studying the impact of OFTP on one's mental problems is not known, which means the 2 years lags between waves may not be true effective interval to detect the lagged associations from time perspective at t‐1 to mental problems at t.

**Figure 1:**
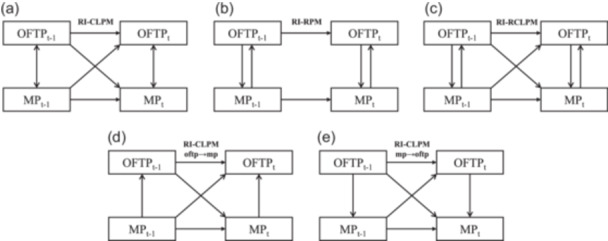
Illustration of the series of structural equation models fit to data on mental health problems (MP) variables and occupational future time perspective (OFTP). (a) Random intercept cross‐lagged panel models, (b) random intercept (contemporaneous) reciprocal without cross‐lagged regressions, (c) random intercept (contemporaneous) reciprocal and cross‐lagged regressions, (d) random intercept (contemporaneous) unidirectional (OFTP → MP) and cross‐lagged regressions, and (e) random intercept unidirectional (MP → OFTP) and cross‐lagged regressions.

**Figure 2:**
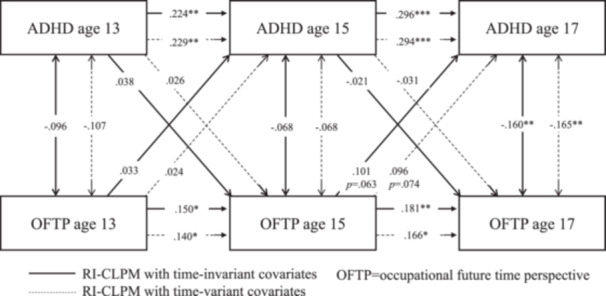
Within‐person associations between occupational future time perspective and ADHD controlling for sex, household income, parental education level, and school type.

**Figure 3:**
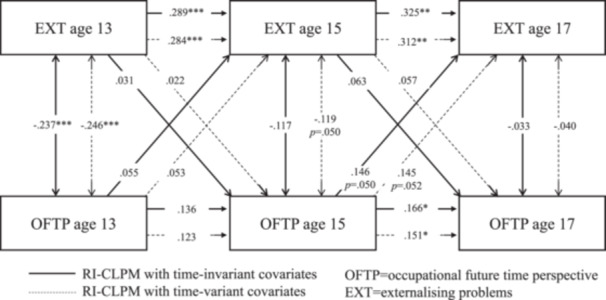
Within‐person associations between occupational future time perspective and externalizing problems controlling for sex, household income, parental education level, and school type.

**Figure 4:**
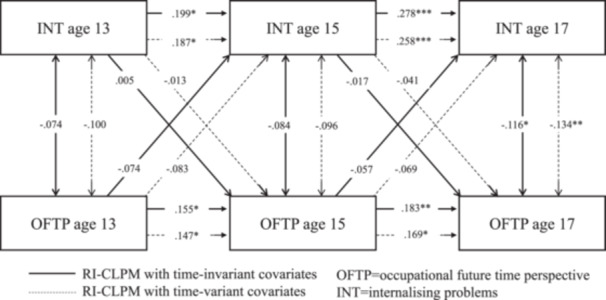
Within‐person associations between occupational future time perspective and internalizing problems controlling for sex, household income, parental education level, and school type.

In light of these considerations to test the robustness of the RI‐CLPMs results, particularly the within‐person cross‐lagged effects, a series of RI‐CLPM variations adapted from Muthen and Asparouhov (2023) and Speyer et al. (2023) were carried out, as illustrated in Figure 1, including: (1) models with (contemporaneous) reciprocal without cross‐lagged regressions (RI‐RPM, No. b in Figure 1), (2) models with (contemporaneous) reciprocal and cross‐lagged regressions (RI‐RCLPM, No. c in Figure 1), (3) models with (contemporaneous) unidirectional (OFTP on mental health problems) and cross‐lagged regressions (RI‐CLPM oftp→mp, No. d in Figure 1), and (4) models with (contemporaneous) unidirectional (mental health problems on OFTP) and cross‐lagged regressions (RI‐CLPM mp→oftp, No. e in Figure 1). The ΔBIC > 10 was again adopted for deciding the optimal model during model comparison, with lower BIC indicating the better model. Considering the possibility that BIC may not meet the standard to favor any model (i.e., ΔBIC < 10), the model parsimony would further be compared to favor the model with fewer parameters.

## 3 RESULTS

### 3.1 Pearson's correlations

Pearson's correlations among all variables are provided in Table S1 in the Supplementary Materials.

### 3.2 RI‐CLPMs

Model fit statistics for the RI‐CLPMs with time‐stable covariate effects (Table S3) and time‐varying covariate effects (Table S10) both met conventional standards for a good fit. Comparisons of treating the effect of the time‐invariant covariates as time‐varying versus stable favored the latter (BIC _vary_ ‐ BIC _stable_: ΔBIC _ADHD_ = 10571.700−10502.786 = 68.914; ΔBIC _EXT_ = 7288.262−7218.624 = 69.638, ΔBIC _INT_ = 9622.051−9563.447 = 58.604); however, both were inspected to check for any major differences by these alternative specifications (Tables S4–S9 models with stable covariates; Tables S11–S16 models with varying covariates; Figures 2, 3, 4).

The RI‐CLPM associations are provided in Figures 2, 3, 4 and Tables S7–S9, with the solid line representing the models controlling for covariate effects on the random intercept and the dotted line representing the models controlling for covariates at each time point. The parameters for all the RI‐CLPMs and their 95% confidence intervals (95% CIs) are described in turn preceding sections.

**ADHD symptoms model** (Table S7): Treating sex, SES and school type as time‐invariant covariates, (i) Autoregressive effects were all positive, which meant adolescents scoring higher than their expected score on ADHD symptoms at one time‐point (based on the temporal mean and random intercept) tended to show higher ADHD symptoms than their expected score at the subsequent age (age 13 → 15: *β* = .224, *p* < .01, [95% CI = 0.078, 0.370]; age 15 → 17: *β* = .296, *p* < .001, [95% CI = 0.165, 0.426]). The same held for OFTP (age 13 → 15: *β* = .150, *p* < .05, [95% CI = 0.016, 0.284]; age 15 → 17: *β* = .181, *p* < .01, [95% CI = 0.045, 0.316]). (ii) There were no significant cross‐lagged effects between OFTP and ADHD symptoms. (iii) Concurrent covariances between OFTP and ADHD symptoms were negative and significant at age 17 (*β* = −.160, *p* < .01, [95% CI = −0.256, −0.064]). Their associations were not significant at ages 13 and 15. (iv) The random intercept factor covariance between ADHD symptoms and OFTP was significant (*r* = −0.614, *p* < .001; [95% CI = −0.835, −0.393]). (v) There were also several significant regressions of the random intercept factors on the covariates: OFTP on sex (*β* = .194, *p* < .001; [95% CI = 0.106, 0.281]), and on school type (*β* = .108, *p* < .05; [95% CI = 0.007, 0.208]); ADHD on sex (*β* = .099, *p* < .05; [95% CI = 0.014, 0.185]), and on parental education level (*β* = .103, *p* < .01; [95% CI = 0.004, 0.203]).

**Externalizing problems model** (Table S8): Treating sex, SES and school type as time‐invariant covariates, (i) Autoregressive effects were all positive and significant for externalizing problems (age 13 → 15: *β* = .289, *p* < .001, [95% CI = 0.155, 0.424]; age 15 → 17: *β* = .325, *p* < .01, [95% CI = 0.125, 0.524]) and for OFTP (age 15 → 17: *β* = .166, *p* < .05, [95% CI = 0.027, 0.304]). (ii) The only significant cross‐lagged effect was a positive small effect for OFTP at age 15 predicting an increase in externalizing problems at age 17 (*β* = .146, *p* = .05, [95% CI = 0.000, 0.292]). (iii) Concurrent covariances between OFTP and externalizing problems were negative and significant at age 13 (*β* = −.237, *p* < .001, [95% CI = −0.338, −0.126]). Their residual associations were not significant at ages 15 and 17. (iv) The random intercept factor covariance between externalizing problems and OFTP was significant (*r* = −0.455, *p* < .001; [95% CI = −0.642, −0.267]. (v) There were also several significant regressions of the random intercept factors on the covariates: OFTP on sex (*β* = .178, *p* < .001, [95% CI = 0.093, 0.263]), and on school type (*β* = .109, *p* < .05, [95% CI = 0.012, 0.207]); and externalizing problems on sex (*β* = −.289, *p* < .001, [95% CI = −0.369, −0.209]), and on school type (*β* = .166, *p* < .001, [95% CI = 0.092, 0.239]).

**Internalizing problems model** (Table S9): Treating sex, SES and school type as time‐invariant covariates, (i) Autoregressive effects were all positive and significant, meaning that adolescents scoring higher than their expected score on internalizing problems (based on the temporal mean and random intercept) tended to show higher internalizing scores than their expected score at the subsequent age (age 13 → 15: *β* = .199, *p* < .05, [95% CI = 0.044, 0.355]; age 15 → 17: *β* = .278, *p* < .001, [95% CI = 0.141, 0.416]). The same effect was observed for OFTP (age 13 → 15: *β* = .155, *p* < .05, [95% CI = 0.022, 0.289]; age 15 → 17: *β* = .183, *p* < .01, [95% CI = 0.048, 0.318]). (ii) Cross‐lagged effects were not significant, which indicated neither OFTP at an earlier age predicted internalizing problems at a later age, nor internalizing problems at an earlier age predicted OFTP at a later age. (iii) Concurrent covariances between OFTP and internalizing problems were negative and significant at age 17 (*β* = .116, *p* < .05, [95% CI = −0.217, −0.015]). Their associations were not significant at age 13 and age 15. (iv) The random intercept factor covariance between internalizing problems and OFTP was significant (*r* = −0.217, *p* < .05, [95% CI = −0.420, −0.013]). (v) There were also several significant regressions of the random intercept factors on the covariates: OFTP on sex (*β* = .204, *p* < .001, [95% CI = 0.116, 0.292]), and school type (*β* = .109, *p* < .05, [95% CI = 0.008, 0.210]); and internalizing problems on sex (*β* = .543, *p* < .001, [95% CI = 0.464, 0.622]).

### 3.3 Sensitivity analysis: Comparison of RI‐CLPMs with alternative models (Tables S17–S19)

**Figure 5:**
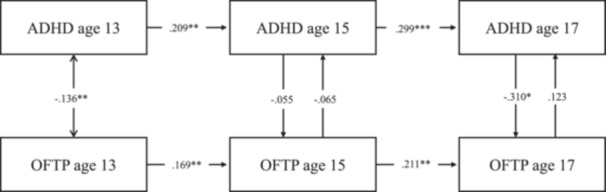
Within‐person associations between occupational future time perspective and ADHD controlling for sex, household income, parents’ highest education level, and school type.

**The comparisons indicated the RI‐RPMs were the optimal models.** The RI‐RPM within‐person effects between OFTP and mental problems are reported as follows (Comprehensive narrative reports for the RI‐RPM results are provided in the Supplementary Materials and in Tables S20–S25):

**ADHD symptoms model** (Table S23, Figure 5): Concurrent covariances between OFTP and ADHD symptoms at age 13 were negative and significant (*β* = −.136, *p* < .01, [95% CI = −0.235, −0.036]). A reciprocal effect was only significant and negative from ADHD to OFTP at age 17 (*β* = −.310, *p* < .05, [95% CI = −0.580, −0.041]).

**Figure 6:**
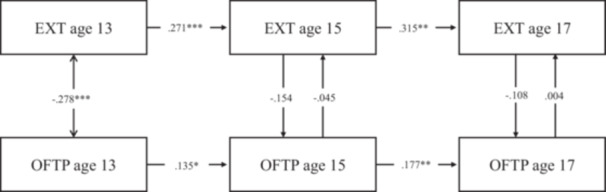
Within‐person associations between occupational future time perspective and externalizing problems controlling for sex, household income, parents’ highest education level, and school type.

**Externalizing problems model** (Table S24, Figure 6): Concurrent covariances between OFTP and externalizing problems at age 13 were negative and significant (*β* = −.278, *p* < .001, [95% CI = −0.369, −0.188]). No reciprocal effects were significant.

**Figure 7:**
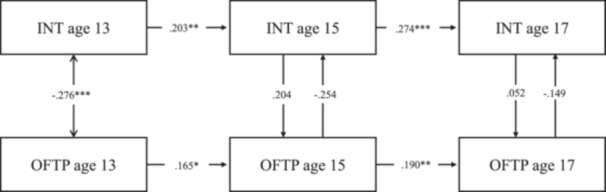
Within‐person associations between occupational future time perspective and internalizing problems controlling for sex, household income, parents’ highest education level, and school type.

**Internalizing problems model** (Table S25, Figure 7): Concurrent covariances between OFTP and internalizing problems at age 13 were not significant. All reciprocal effects were not significant. The random intercept factor covariance between internalizing problems and OFTP was significant (*r* = −0.276, *p* < .001; [95% CI = −0.418, −0.133]).

## 4 DISCUSSION

Our study represents one of the first attempts to explore the role of OFTP in mental health from an intra‐individual developmental perspective. Specifically, we focus on the occupational time perspective and its relationship with internalizing, externalizing, and ADHD symptoms. Building upon previous research, which has suggested negative associations between OFTP and various mental health problems, and considering the incorporation of OFTP into interventions, our study endeavors to investigate whether such associations exist at the within‐person level across the developmental span of ages 13–17. Furthermore, we examined the possibility of reverse effects from mental health symptoms to OFTP over the same developmental period. Our findings suggested negative associations between OFTP and specific mental health problems at both the between‐person and concurrent within‐person levels (i.e., OFTP with ADHD at age 17, externalizing problems at age 13, and internalizing problems at age 17), which is consistent with previous cross‐sectional findings (Kooij et al., 2018; Stolarski et al., 2016; Weissenberger et al., 2020), there was little robust evidence for any prospective temporal within‐person effects.

We did not observe any significant cross‐lagged predictions between OFTP and mental health problems at within‐person level. Notably, only a small effect emerged from higher OFTP at age 15 to increased externalizing problems at age 17 at the within‐person level (*β* = .146, *p* = .05; [95% CI = 0.000, 0.292]) in a RI‐CLPM. In sensitivity analyses using (contemporaneous) reciprocal without cross‐lagged regressions (RI‐RPM), the only significant prospective within‐person effect was ADHD negatively predicting OFTP at age 17 (*β* = −.310, *p* = .024, [95% CI = −0.580, −0.041]). The lack of cross‐lagged effect between age 13 and 15 could be explained by the fact that a career might still seem distant. However, the only significant reciprocal effect in a RI‐RPM model (Figure 5) suggested that at age 17, adolescents high above their own individual average ADHD symptoms in the past 12 months had more difficulty having a positive OFTP. This finding is consistent with the neural processing abnormality of ADHD on time (e.g., Hart et al., 2012; Mette, 2023), and Scholtens et al.'s (2013) finding that ADHD symptoms in 11th grade negatively affected future orientation in 12th grade.

Two potential explanations arise for the little within‐person evidence yielded by the present study. Firstly, this finding may suggest that the negative associations observed in existing cross‐sectional studies may primarily reflect interindividual differences (e.g., Kooij et al., 2018; Stolarski et al., 2016; Weissenberger et al., 2020). Some other associations found in existing longitudinal studies also lack discernible associations evident at the intra‐individual level between a general FTP and mental health (Konowalczyk et al., 2018), internet gaming disorder (Lukavská, 2018) and posttraumatic stress disorder (Jung et al., 2021). This underscores the importance of investigating direct within‐person associations between OFTP and mental health issues, an aspect often overlooked in prior longitudinal studies. Therefore, the present findings indicate that enhancing OFTP may contribute little to achieving better mental health, providing insights for school interventional practices.

Secondly, the 2‐year lag between the data waves may not be optimal for detecting within‐person causal effects of OFTP on mental health problems, or for monitoring future aspirations to activate sustained self‐regulation. Based on the significant within‐time point associations between these constructs observed across model variations in our study, shorter time lags may be advisable. Experimental research employing future thinking techniques typically evaluates relevant outcomes, such as impulsivity (measured via delay discounting tasks, a transdiagnostic marker; Amlung et al., 2019), immediately post‐manipulation (delay discounting task, a transdiagnostic marker; Amlung et al., 2019) immediately after manipulation (e.g., within a session, Rung & Madden, 2018; 1‐week interval, Fieulaine & Martinez, 2012). An alternative explanation might entail augmenting the frequency of future aspiration monitoring. The current level of monitoring regarding future occupational goals may not sufficiently engage self‐regulatory processes. This proposition is supported by a meta‐analysis of randomized controlled trials (RCTs) utilizing goal monitoring strategies, which indicated that the frequency of goal monitoring influenced the attainment of intervention outcomes (k = 138, *N* = 19,951; Harkin et al., 2016). Therefore, future studies incorporating shorter time lags or higher monitoring frequencies are promising to explore this topic.

### 4.1 Contributions, limitations and future directions

Our study elucidated that OFTP, specifically concerning future occupation and the link between current education and future career prospects, did not exert a significant influence on symptoms of ADHD, internalizing, and externalizing behaviors, as evidenced by within‐person analysis. This clarification holds significance for school‐based mental health education and intervention practices. Clinical interventions typically comprise a comprehensive package of elements, posing challenges in discerning the active components. Our findings suggest that emphasizing occupational aspirations may not be a crucial component in mental health interventions. In combination with existing research, strategies focused on the problem domain related expectations may activate more operationalizable self‐regulation, especially when the envisioned future directly addresses the domain of mental health issues such as concrete expectations for reducing substance use could be more effective (e.g., Matischek‐Jauk et al., 2018). Similarly, relying solely on OFTP training within school‐based future perspective education may not be a universally applicable solution for educational settings, nor is it likely to be adequate for fostering holistic student development.

Several limitations warrant acknowledgment. First, the SBQ utilized in this study is not a diagnostic tool for mental health problems, and our examination was confined to symptoms of ADHD, aggression, depression, and anxiety, thus limiting the scope of our findings to these specific domains. Second, the measurement of OFTP was based on only three items. Future investigations could benefit from employing more comprehensive measures and incorporating clinical samples to further explore the temporal covariances across adolescence. Third, the time lags between waves in our study may not have been optimal for detecting associations between time perspective and mental health. Future research could consider exploring these relations using various shorter time lags, such as through ecological momentary assessment designs spanning days, weeks, or months, to better capture the entirety of adolescence. Fourth, only mental problems (ADHD, externalizing, internalizing problems) were worded as temporally ahead of OFTP (12 months, 1 month), but not in reverse. This unidirectional temporality could possibly be the reason for no significant prediction from OFTP to mental health problems. If OFTP took place before the mental health problems with a short interval (e.g., 1 week) between waves, it may likely be more optimal to examine the lagged within‐person effect. Fifth, we did not analyze the potential mechanisms linking OFTP to mental health for the significant between‐person associations identified. Future research will be necessary to clarify the underlying mechanism between OFTP and mental health at this level, as well as its absence at the within‐person level. Sixth, family income was only assessed at baseline (age 7), potentially overlooking changes in socioeconomic status during adolescence. Subsequent studies could consider collecting contemporary socioeconomic data at each wave alongside other variables to examine how changes may influence within‐person covariance. Seventh, the findings were based on a sample of the typical adolescent population in a well‐developed country, replications are necessary among related clinical populations and in less developed countries and areas. Lastly, other developmental trajectory models, or approaches that enable causal inference in longitudinal observational data, such as counterfactual analysis, should be considered.

## 5 CONCLUSION

The current study investigated the reciprocal developmental dynamics between OFTP and adolescents’ internalizing, externalizing, and ADHD symptoms. While our findings confirmed a promotive role of OFTP for mental health at the between‐group and concurrent within‐person levels, they also suggested that simply enhancing an adolescent's OFTP may not directly lead to improvements in their mental health at the temporal within‐person level. Consequently, our results suggest that including OFTP as a standalone intervention component aimed at improving mental health outcomes in adolescents may not be effective. Further research and replication are necessary to confirm these results.

## CONFLICT OF INTEREST STATEMENT

The authors declare no conflict of interest.

## ETHICS STATEMENT

Consistent with the principles of the Declaration of Helsinki, ethical approval of the z‐proso study was acquired from the Ethics Committee at the Faculty of Arts and Social Sciences of the University of Zurich. Before the investigation, active informed consent was signed by the adolescents themselves from age 13. Additionally, guardians had the option to withdraw their children from the study until the children reached the age of 18.

## Supporting information

Supporting information.

## Data Availability

The data that support the findings of this study are available on request from the corresponding author. The data are not publicly available due to privacy or ethical restrictions. The data supporting the present study can be accessed by requesting it from the z‐proso program director, with consideration for protecting participants’ privacy. Code is provided by the first author upon request.
