# Supplementary material for: Occupational future time perspective and mental health problems across adolescence: Random‐intercept cross‐lagged panel analysis and alternative variations
Source: J Adolesc. 2024 Oct 22;97(2):526–39. doi: 10.1002/jad.12438 (PMC11791734; doi:10.1002/jad.12438)
Supplement: Supplementary file 1 — Supporting information. [file JAD-97-526-s001.docx]

**Supplementary materials**

| **Table S1. Pearson's Correlations among all variables** | | | | | | | | | | | | | | | |
| --- | --- | --- | --- | --- | --- | --- | --- | --- | --- | --- | --- | --- | --- | --- | --- |
|  | Sex | Income | EduMax | SType | TP5 | TP6 | TP7 | ADHD5 | ADHD6 | ADHD7 | AGGR5 | AGGR6 | AGGR7 | INTER5 | INTER6 |
| Income | 0.008 | _ |  |  |  |  |  |  |  |  |  |  |  |  |  |
| EduMax | -0.021 | .377^**^ | _ |  |  |  |  |  |  |  |  |  |  |  |  |
| SType | -.052^*^ | -.311^**^ | -.426^**^ | _ |  |  |  |  |  |  |  |  |  |  |  |
| TP5 | .087^**^ | -0.009 | 0.012 | -0.003 | _ |  |  |  |  |  |  |  |  |  |  |
| TP6 | .099^**^ | -0.001 | -.063^*^ | .087^**^ | .432^**^ | _ |  |  |  |  |  |  |  |  |  |
| TP7 | .171^**^ | -.079^*^ | -.088^**^ | .125^**^ | .364^**^ | .459^**^ | _ |  |  |  |  |  |  |  |  |
| ADHD5 | 0.005 | .071^*^ | .081^**^ | -0.052 | -.267^**^ | -.178^**^ | -.171^**^ | _ |  |  |  |  |  |  |  |
| ADHD6 | .073^**^ | 0.016 | .103^**^ | -.110^**^ | -.194^**^ | -.258^**^ | -.240^**^ | .476^**^ | _ |  |  |  |  |  |  |
| ADHD7 | .085^**^ | 0.050 | 0.053 | -0.055 | -.171^**^ | -.149^**^ | -.274^**^ | .379^**^ | .527^**^ | _ |  |  |  |  |  |
| AGGR5 | -.231^**^ | -.072^*^ | -.081^**^ | .135^**^ | -.313^**^ | -.144^**^ | -.127^**^ | .349^**^ | .171^**^ | .192^**^ | _ |  |  |  |  |
| AGGR6 | -.187^**^ | -.075^*^ | -.087^**^ | .119^**^ | -.203^**^ | -.236^**^ | -.166^**^ | .174^**^ | .317^**^ | .232^**^ | .548^**^ | _ |  |  |  |
| AGGR7 | -.184^**^ | -.078^*^ | -.082^**^ | .124^**^ | -.171^**^ | -.095^**^ | -.195^**^ | .161^**^ | .201^**^ | .342^**^ | .466^**^ | .594^**^ | _ |  |  |
| INTER5 | .282^**^ | 0.034 | 0.028 | -.066^*^ | -.113^**^ | -.079^**^ | -0.019 | .345^**^ | .307^**^ | .252^**^ | .238^**^ | .154^**^ | .173^**^ | _ |  |
| INTER6 | .395^**^ | 0.006 | 0.008 | -.059^*^ | -.065^*^ | -.084^**^ | -0.016 | .225^**^ | .373^**^ | .313^**^ | .078^**^ | .180^**^ | .129^**^ | .541^**^ | _ |
| INTER7 | .401^**^ | 0.014 | -0.031 | -0.016 | -.068^*^ | -0.052 | -.068^*^ | .188^**^ | .271^**^ | .437^**^ | .059^*^ | .095^**^ | .171^**^ | .450^**^ | .614^**^ |

*Note.* Sex (1=male; 2=female); Income=household income (1= 0- 1999\Monat ODER 0- 23999\Jahr; 10= 15000- mehr\Monat ODER 180000- mehr\Jahr); EduMax=parents’ education level (1= incomplete compulsory school; 10= university, Swiss Federal Institute of Technology); SType=school type (0=gymnasium; 1=occupation); TP=future time perspective; EXT=externalising problems, INT=internalising problems; **p*<.05; ***p*<.01.

| **Table S2**. Sample size with valid data from at least 1, 2, or 3 time points on each construct | | | |
| --- | --- | --- | --- |
| **Control Sex, SES, School Type** | **ADHD** | **Externalising** | **Internalising** |
| At least 1 time points | 970 | 970 | 969 |
| At least 2 time points | 903 | 903 | 903 |
| 3 time points | 890 | 890 | 890 |

**Section 1:**

**RI-CLPMs results with sex, SES (household income, Parents’ education level), and school type**

**as time-stable covariates**

**Table S3.** Model fit statistics for models controlling for sex, SES (household income, parents’ highest education level), and school type as covariates with time-stable effects.

| **Models controlling for sex,** **SES,**  **and school type** | **CFI** | **TLI** | **RMSEA** | **SRMR** |
| --- | --- | --- | --- | --- |
| Future time perspective and ADHD | .975 | .944 | .040 | .025 |
| Future time perspective and EXT | .978 | .949 | .041 | .027 |
| Future time perspective and INT | .970 | .931 | .048 | .036 |

*Note.* FTP= future time perspective; EXT=externalising problems; INT=internalising problems.

The between-person and within-person correlations among OFTP, mental health problems and covariates are also provided in S4-S6. There were significant between-person correlations for the control variables (Tables S4-S6), indicating the significant associations between these variables and OFTP and mental health: i) female was correlated with significantly higher OFTP, ADHD symptoms, and internalising problems, while male was correlated with significantly higher externalising problems; ii) lower household income was correlated with higher externalising problems; iii) lower parental educational level was correlated with lower ADHD symptoms, but higher externalising problems; and iv) occupational education was correlated with lower ADHD symptoms and internalising problems, but higher externalising problems.

**Table S4.** Correlations controlling for sex, SES (household income, parents’ highest education level), and school type among future time perspective and ADHD at the between- and within-person levels

|  | 1 | 2 | 6 | 7 | 8 | 9 | 10 | 11 |
| --- | --- | --- | --- | --- | --- | --- | --- | --- |
| **Between-person correlations** |  |  |  |  |  |  |  |  |
| 1. RI_FTP | _ |  |  |  |  |  |  |  |
| 2. RI_ADHD | -.580*** | _ |  |  |  |  |  |  |
| 3. Sex | .189*** | .101* | _ | _ | _ | _ | _ | _ |
| 4. Household income | -.041 | .063 | _ | _ | _ | _ | _ | _ |
| 5. Parents’ education level | -.032 | .128** | _ | _ | _ | _ | _ | _ |
| 6. School type | .095* | -.103* | _ | _ | _ | _ | _ | _ |
| **Within-person correlations** |  |  |  |  |  |  |  |  |
| 6. Age 13 FTP | _ | _ | _ |  |  |  |  |  |
| 7. Age 15 FTP | _ | _ | .147* | _ |  |  |  |  |
| 8. Age 17 FTP | _ | _ | .026 | .182** | _ |  |  |  |
| 9. Age 13 ADHD | _ | _ | -.096 | .023 | .00 | _ |  |  |
| 10. Age 15 ADHD | _ | _ | .012 | -.055 | -.031 | .220** | _ |  |
| 11. Age 17 ADHD | _ | _ | .018 | .084 | -.140* | .068* | .290*** | _ |

*Note.* RI*=*Random-Intercept; FTP= future time perspective; **p*<.05; ***p*<.01; ****p*<.001.

**Table S5.** Correlations controlling for sex, SES (household income, parents’ highest education level), and school type among future time perspective and externalising problems at the between- and within-person levels

|  | 1 | 2 | 6 | 7 | 8 | 9 | 10 | 11 |
| --- | --- | --- | --- | --- | --- | --- | --- | --- |
| **Between-person correlations** |  |  |  |  |  |  |  |  |
| 1. RI_FTP | _ |  |  |  |  |  |  |  |
| 2. RI_EXT | -.446*** | _ |  |  |  |  |  |  |
| 3. Sex | .173*** | -.295*** | _ | _ | _ | _ | _ | _ |
| 4. Household income | -.043 | -.121** | _ | _ | _ | _ | _ | _ |
| 5. Parents’ education level | -.035 | -.116** | _ | _ | _ | _ | _ | _ |
| 6. School type | .099* | .207*** | _ | _ | _ | _ | _ | _ |
| **Within-person correlations** |  |  |  |  |  |  |  |  |
| 6. Age 13 FTP | _ | _ | _ |  |  |  |  |  |
| 7. Age 15 FTP | _ | _ | .128 | _ |  |  |  |  |
| 8. Age 17 FTP | _ | _ | .020 | .159* | _ |  |  |  |
| 9. Age 13 EXT | _ | _ | -.237*** | -.001 | .017 | _ |  |  |
| 10. Age 15 EXT | _ | _ | -.014 | -.104 | .045 | .276*** | _ |  |
| 11. Age 17 EXT | _ | _ | .014 | .112 | .007 | .090 | .309** | _ |

*Note.* RI*=*Random-Intercept; FTP= future time perspective; EXT=externalising problems; **p*<.05; ***p*<.01; ****p*<.001.

**Table S6.** Correlations controlling for sex, SES (household income, parents’ highest education level), and school type among future time perspective and internalising problems at the between- and within-person levels

|  | 1 | 2 | 6 | 7 | 8 | 9 | 10 | 11 |
| --- | --- | --- | --- | --- | --- | --- | --- | --- |
| **Between-person correlations** |  |  |  |  |  |  |  |  |
| 1. RI_FTP | _ |  |  |  |  |  |  |  |
| 2. RI_INT | -.074 | _ |  |  |  |  |  |  |
| 3. Sex | .199*** | .545*** | _ | _ | _ | _ | _ | _ |
| 4. Household income | -.045 | .044 | _ | _ | _ | _ | _ | _ |
| 5. Parents’ education level | -.035 | .027 | _ | _ | _ | _ | _ | _ |
| 6. School type | .098* | -.079* | _ | _ | _ | _ | _ | _ |
| **Within-person correlations** |  |  |  |  |  |  |  |  |
| 6. Age 13 FTP | _ | _ | _ |  |  |  |  |  |
| 7. Age 15 FTP | _ | _ | .155* | _ |  |  |  |  |
| 8. Age 17 FTP | _ | _ | .030 | .185** | _ |  |  |  |
| 9. Age 13 INT | _ | _ | -.074 | -.007 | -.005 | _ |  |  |
| 10. Age 15 INT | _ | _ | -.089 | -.094 | -.034 | .205* | _ |  |
| 11. Age 17 INT | _ | _ | -.034 | -.083 | -.129* | .057 | .284*** | _ |

*Note.* RI*=*Random-Intercept; FTP= future time perspective; INT=internalising problems; **p*<.05; ***p*<.01; ****p*<.001.

**Table S7.** RI-CLPM standardised parameters for future time perspective and ADHD controlling for sex, SES (household income, parents’ highest education level), and school type as covariates with time-stable effects

| **M1: ADHD Model controlling for sex, SES, and School type at random intercept** (BIC = 10502.786) | | | |
| --- | --- | --- | --- |
| **Within-person parameters:** | **Estimate [95%CI]** | **SE** | ***p*** |
| *Autoregressive parameters* |  |  |  |
| FTP age 15 → FTP age 17 | .181 [.045, .316] | .069 | .009 |
| FTP age 13 → FTP age 15 | .150 [.016, .284] | .068 | .028 |
| ADHD age 15 → ADHD age 17 | .296 [.165, .426] | .067 | <.001 |
| ADHD age 13 → ADHD age 15 | .224 [.078, .370] | .075 | .003 |
| *Cross-lagged parameters* |  |  |  |
| ADHD age 15 → FTP age 17 | -.021 [-.140, .098] | .061 | .730 |
| ADHD age 13 → FTP age 15 | .038 [-.081, .157] | .061 | .534 |
| FTP age 15 → ADHD age 17 | .101 [-.006, .207] | .054 | .063 |
| FTP age 13 → ADHD age 15 | .033 [-.078, .145] | .057 | .559 |
| *Within-time parameters* |  |  |  |
| FTP age 17 with ADHD age 17 | -.160 [-.256, -.064] | .049 | .001 |
| FTP age 15 with ADHD age 15 | -.068 [-.181, .045] | .058 | .239 |
| FTP age 13 with ADHD age 13 | -.096 [-.223, .030] | .064 | .135 |
| **Between-person parameter:** |  |  |  |
| FTP with ADHD | -.614 [-.835, -.393] | .113 | <.001 |
| *Covariates* |  |  |  |
| Sex→ FTP | .194 [.106, .281] | .04 | < .001 |
| Income→ FTP | -.013 [-.104, .078] | .05 | .746 |
| Parents’ education→ FTP | .020 [-.078, .118] | .05 | .480 |
| School type→ FTP | .108 [.007, .208] | .051 | .036 |
| Sex→ ADHD | .099 [.014, .185] | .044 | .023 |
| Income→ ADHD | .007 [-.085, .099] | .047 | .888 |
| Parents’ education→ ADHD | .103 [.004, .203] | .051 | .042 |
| School type→ ADHD | -.053 [-.150, .045] | .050 | .290 |

*Note.* FTP= future time perspective. The within-person parameters include autoregressive, cross-lagged, and within-time parameters.

**Table S8.** RI-CLPM standardised parameters for future time perspective and externalising problems controlling for sex, SES (household income, parents’ highest education level), and school type as covariates with time-stable effects

| **M2: EXT Model controlling for sex, SES, and School type at random intercept** (BIC = 7218.624) | | | |
| --- | --- | --- | --- |
| **Within-person parameters:** | **Estimate [95%CI]** | **SE** | ***p*** |
| *Autoregressive parameters* |  |  |  |
| FTP age 15 → FTP age 17 | .166 [.027, .304] | .071 | .019 |
| FTP age 13 → FTP age 15 | .136 [-.001, .272] | .069 | .051 |
| EXT age 15 → EXT age 17 | .325 [.125, .524] | .102 | .001 |
| EXT age 13 → EXT age 15 | .289 [.155, .424] | .069 | <.001 |
| *Cross-lagged parameters* |  |  |  |
| EXT age 15 → FTP age 17 | .063 [-.066, .191] | .066 | .341 |
| EXT age 13 → FTP age 15 | .031 [-.094, .157] | .064 | .624 |
| FTP age 15 → EXT age 17 | .146 [.000, .292] | .074 | .050 |
| FTP age 13 → EXT age 15 | .055 [-.052, .161] | .054 | .312 |
| *Within-time parameters* |  |  |  |
| FTP age 17 with EXT age 17 | -.033 [-.166, .100] | .068 | .628 |
| FTP age 15 with EXT age 15 | -.117 [-.235, .002] | .060 | .054 |
| FTP age 13 with EXT age 13 | -.237 [-.338, -.126] | .051 | <.001 |
| **Between-person parameter:** |  |  |  |
| FTP with EXT | -.455 [-.642, -.267] | .096 | <.001 |
| *Covariates* |  |  |  |
| Sex→ FTP | .178 [.093, .263] | .043 | < .001 |
| Income→ FTP | -.013 [-.102, .076] | .045 | .768 |
| Parents’ education→ FTP | .018 [-.077, .113] | .048 | .710 |
| School type→ FTP | .109 [.012, .207] | .050 | .029 |
| Sex→ EXT | -.289 [-.369, -.209] | .041 | < .001 |
| Income→ EXT | -.060 [-.141, .021] | .041 | .143 |
| Parents’ education→ EXT | -.024 [-.110, .062] | .044 | .583 |
| School type→ EXT | .166 [.092, .239] | .038 | < .001 |

*Note.* FTP= future time perspective; EXT=externalising problems. The within-person parameters include autoregressive, cross-lagged, and within-time parameters.

**Table S9.** RI-CLPM standardised parameters for future time perspective and internalising problems controlling for sex, SES (household income, parents’ highest education level), and school type as covariates with time-stable effects

| **M3: INT Model controlling for sex, SES, and School type at random intercept** (BIC = 9563.447) | | | |
| --- | --- | --- | --- |
| **Within-person parameters:** | **Estimate [95% CI]** | **SE** | ***p*** |
| *Autoregressive parameters* |  |  |  |
| FTP age 15 → FTP age 17 | .183 [.048, .318] | .069 | .008 |
| FTP age 13 → FTP age 15 | .155 [.022, .289] | .068 | .022 |
| INT age 15 → INT age 17 | .278 [.141, .416] | .070 | <.001 |
| INT age 13 → INT age 15 | .199 [.044, .355] | .079 | .012 |
| *Cross-lagged parameters* |  |  |  |
| INT age 15 → FTP age 17 | -.017 [-.132, .098] | .059 | .771 |
| INT age 13 → FTP age 15 | .005 [-.123, .133] | .065 | .942 |
| FTP age 15 → INT age 17 | -.057 [-.161, .048] | .053 | .289 |
| FTP age 13 → INT age 15 | -.074 [-.187, .039] | .058 | .197 |
| *Within-time parameters* |  |  |  |
| FTP age 17 with INT age 17 | -.116 [-.217, -.015] | .052 | .025 |
| FTP age 15 with INT age 15 | -.084 [-.192, .023] | .055 | .124 |
| FTP age 13 with INT age 13 | -.074 [-.194, .045] | .061 | .224 |
| **Between-person parameter:** |  |  |  |
| FTP with INT | -.217 [-.420, -.013] | .104 | .037 |
| *Covariates* |  |  |  |
| Sex→ FTP | .204 [.116, .292] | .045 | < .001 |
| Income→ FTP | -.017 [-.108, .075] | .047 | .722 |
| Parents’ education→ FTP | .019 [-.080, .117] | .050 | .708 |
| School type→ FTP | .109 [.008, .210] | .052 | .035 |
| Sex→ INT | .543 [.464, .622] | .040 | < .001 |
| Income→ INT | .034 [-.043, .111] | .039 | .387 |
| Parents’ education→ INT | -.003 [-.087, .082] | .043 | .952 |
| School type→ INT | -.047 [-.126, .033] | .041 | .250 |

*Note.* FTP= future time perspective; INT=internalising problems. The within-person parameters include autoregressive, cross-lagged, and within-time parameters.

**Section 2:**

**RI-CLPMs results with sex, SES (household income, parent education level), and school type**

**as covariates having time-varying effects**

**Table S10.** Model fit statistics for models controlling for sex, SES (household income, parents’ highest education level), and school type as covariates with time-varying effects at each time point.

| **Models controlling for sex,** **SES, and school type** | **CFI** | **TLI** | **RMSEA** | **SRMR** |
| --- | --- | --- | --- | --- |
| Future time perspective and ADHD | .999 | .967 | .031 | .008 |
| Future time perspective and EXT | .999 | .965 | .034 | .008 |
| Future time perspective and INT | .999 | .942 | .044 | .007 |

*Note.* FTP= future time perspective; EXT=externalising problems; INT=internalising problems.

**Table S11.** Correlations controlling for sex, SES (household income, parents’ highest education level), and school type among future time perspective and ADHD at the between- and within-person levels

|  | 1 | 2 | 6 | 7 | 8 | 9 | 10 | 11 |
| --- | --- | --- | --- | --- | --- | --- | --- | --- |
| **Between-person correlations** |  |  |  |  |  |  |  |  |
| 1. RI_FTP | _ |  |  |  |  |  |  |  |
| 2. RI_ADHD | -.586*** | _ |  |  |  |  |  |  |
| 3. Sex | _ | _ | .065* | .094** | .173*** | .009 | .088** | .081* |
| 4. Household income | _ | _ | -.011 | .002 | -.060 | .063 | .010 | .040 |
| 5. Parents’ education level | _ | _ | .037 | -.035 | -.060 | .068* | .097** | .065 |
| 6. School type | _ | _ | -.016 | .084* | .099** | -.034 | -.092** | -.063 |
| **Within-person correlations** |  |  |  |  |  |  |  |  |
| 6. Age 13 FTP | _ | _ | _ |  |  |  |  |  |
| 7. Age 15 FTP | _ | _ | .137* | _ |  |  |  |  |
| 8. Age 17 FTP | _ | _ | .023 | .168* | _ |  |  |  |
| 9. Age 13 ADHD | _ | _ | -.107 | .011 | -.005 | _ |  |  |
| 10. Age 15 ADHD | _ | _ | -.001 | -.060 | -.041 | .227** | _ |  |
| 11. Age 17 ADHD | _ | _ | .013 | .078 | -.151* | .068* | .289*** | _ |

*Note.* RI*=*Random-Intercept; FTP= future time perspective; **p*<.05; ***p*<.01; ****p*<.001.

**Table S12.** Correlations controlling for sex, SES (household income, parents’ highest education level), and school type among future time perspective and externalising problems at the between- and within-person levels

|  | 1 | 2 | 6 | 7 | 8 | 9 | 10 | 11 |
| --- | --- | --- | --- | --- | --- | --- | --- | --- |
| **Between-person correlations** |  |  |  |  |  |  |  |  |
| 1. RI_FTP | _ |  |  |  |  |  |  |  |
| 2. RI_EXT | -.436*** | _ |  |  |  |  |  |  |
| 3. Sex | _ | _ | .064* | .095** | .176*** | -.230*** | -.181*** | -.187*** |
| 4. Household income | _ | _ | -.011 | -.001 | -.062 | -.078* | -.081* | -.084* |
| 5. Parents’ education level | _ | _ | .037 | -.035 | -.059 | -.102* | -.066* | -.079* |
| 6. School type | _ | _ | -.015 | .083* | .100** | .156*** | .144*** | .142*** |
| **Within-person correlations** |  |  |  |  |  |  |  |  |
| 6. Age 13 FTP | _ | _ | _ |  |  |  |  |  |
| 7. Age 15 FTP | _ | _ | .118 | _ |  |  |  |  |
| 8. Age 17 FTP | _ | _ | .017 | .145* | _ |  |  |  |
| 9. Age 13 EXT | _ | _ | -.246*** | -.008 | .014 | _ |  |  |
| 10. Age 15 EXT | _ | _ | -.017 | -.110 | .104 | .271*** | _ |  |
| 11. Age 17 EXT | _ | _ | .012 | .111 | -.003 | .085 | .300** | _ |

*Note.* RI*=*Random-Intercept; FTP= future time perspective; EXT=externalising problems; **p*<.05; ***p*<.01; ****p*<.001.

**Table S13.** Correlations controlling for sex, SES (household income, parents’ highest education level), and school type among future time perspective and internalising problems at the between- and within-person levels

|  | 1 | 2 | 6 | 7 | 8 | 9 | 10 | 11 |
| --- | --- | --- | --- | --- | --- | --- | --- | --- |
| **Between-person correlations** |  |  |  |  |  |  |  |  |
| 1. RI_FTP | _ |  |  |  |  |  |  |  |
| 2. RI_INT | -.178 | _ |  |  |  |  |  |  |
| 3. Sex | _ | _ | .064 | .094** | .173*** | .286*** | .402*** | .402*** |
| 4. Household income | _ | _ | -.010 | -.002 | -.061 | .049 | .022 | .013 |
| 5. Parents’ education level | _ | _ | .036 | -.035 | -.062 | .027 | .018 | .005 |
| 6. School type | _ | _ | -.015 | .084* | .099** | -.062* | -.051 | -.041 |
| **Within-person correlations** |  |  |  |  |  |  |  |  |
| 6. Age 13 FTP | _ | _ | _ |  |  |  |  |  |
| 7. Age 15 FTP | _ | _ | .148* | _ |  |  |  |  |
| 8. Age 17 FTP | _ | _ | .029 | .173* | _ |  |  |  |
| 9. Age 13 INT | _ | _ | -.100 | -.028 | -.013 | _ |  |  |
| 10. Age 15 INT | _ | _ | -.102 | -.111 | -.059 | .195* | _ |  |
| 11. Age 17 INT | _ | _ | -.036 | -.097 | -.154* | .052 | .265*** | _ |

*Note.* RI*=*Random-Intercept; FTP= future time perspective; INT=internalising problems; **p*<.05; ***p*<.01; ****p*<.001.

**Table S14.** RI-CLPM standardised parameters for future time perspective and ADHD controlling for sex, SES (household income, parents’ highest education level), and school type as covariates with time-varying effects

| **M4: ADHD Model controlling for sex, SES and school type at each time point** (BIC = 10571.700) | | | | | |
| --- | --- | --- | --- | --- | --- |
| **Within-person parameters:** | | **Estimate** | **SE** | | ***p*** |
| *Autoregressive parameters* | |  |  | |  |
| FTP age 15 → FTP age 17 | | .166 | .069 | | .016 |
| FTP age 13 → FTP age 15 | | .140 | .068 | | .041 |
| ADHD age 15 → ADHD age 17 | | .294 | .067 | | <.001 |
| ADHD age 13 → ADHD age 15 | | .229 | .074 | | .002 |
| *Cross-lagged parameters* | |  |  | |  |
| ADHD age 15 → FTP age 17 | | -.031 | .061 | | .611 |
| ADHD age 13 → FTP age 15 | | .026 | .061 | | .672 |
| FTP age 15 → ADHD age 17 | | .096 | .054 | | .074 |
| FTP age 13 → ADHD age 15 | | .024 | .057 | | .675 |
| *Within-time parameters* | |  |  | |  |
| FTP age 17 with ADHD age 17 | | -.165 | .049 | | .001 |
| FTP age 15 with ADHD age 15 | | -.068 | .058 | | .235 |
| FTP age 13 with ADHD age 13 | | -.107 | .064 | | .094 |
| **Between-person parameter:** | |  |  | |  |
| FTP with ADHD | | -.586 | .106 | | <.001 |
| *Covariates* | |  |  | |  |
| Sex→ FTP age 13 | | .065 | .033 | | .046 |
| Sex→ FTP age 15 | | .098 | .032 | | .002 |
| Sex→ FTP age 17 | | .177 | .033 | | <.001 |
| Sex→ ADHD age 13 | | .009 | .033 | | .774 |
| Sex→ ADHD age 15 | | .086 | .031 | | .006 |
| Sex→ ADHD age 17 | | .079 | .033 | | .016 |
| Income→ FTP age 13 | -.029 | | | .035 | .404 |
| Income→ FTP age 15 | .031 | | | .034 | .360 |
| Income→ FTP age 17 | -.025 | | | .037 | .490 |
| Income→ ADHD age 13 | .044 | | | .036 | .219 |
| Income→ ADHD age 15 | -.044 | | | .036 | .224 |
| Income→ ADHD age 17 | .012 | | | .037 | .746 |
| Parents’ education→ FTP age 13 | .048 | | | .037 | .199 |
| Parents’ education→ FTP age 15 | -.005 | | | .037 | .881 |
| Parents’ education→ FTP age 17 | -.009 | | | .038 | .824 |
| Parents’ education→ ADHD age 13 | .052 | | | .038 | .166 |
| Parents’ education→ ADHD age 15 | .086 | | | .036 | .016 |
| Parents’ education→ ADHD age 17 | .046 | | | .039 | .236 |
| School type→ FTP age 13 | -.002 | | | .037 | .959 |
| School type→ FTP age 15 | .095 | | | .036 | .009 |
| School type→ FTP age 17 | .095 | | | .037 | .011 |
| School type→ ADHD age 13 | .003 | | | .036 | .932 |
| School type→ ADHD age 15 | -.066 | | | .035 | .056 |
| School type→ ADHD age 17 | -.036 | | | .036 | .312 |

*Note.* FTP= future time perspective. The within-person parameters include autoregressive, cross-lagged, and within-time parameters.

**Table S15.** RI-CLPM standardised parameters for future time perspective and externalising problems controlling for sex, SES (household income, parents’ highest education level), and school type as covariates with time-varying effects

| **M5: EXT Model controlling for sex, SES and school type at each time point** (BIC = 7288.262) | | | | | | | | |
| --- | --- | --- | --- | --- | --- | --- | --- | --- |
| **Within-person parameters:** | **Estimate** | | | | | **SE** | ***p*** | |
| *Autoregressive parameters* |  | | | | |  |  | |
| FTP age 15 → FTP age 17 | .151 | | | | | .070 | .032 | |
| FTP age 13 → FTP age 15 | .123 | | | | | .070 | .078 | |
| EXT age 15 → EXT age 17 | .312 | | | | | .103 | .002 | |
| EXT age 13 → EXT age 15 | .284 | | | | | .068 | <.001 | |
| *Cross-lagged parameters* |  | | | | |  |  | |
| EXT age 15 → FTP age 17 | .057 | | | | | .067 | .395 | |
| EXT age 13 → FTP age 15 | .022 | | | | | .064 | .733 | |
| FTP age 15 → EXT age 17 | .145 | | | | | .075 | .052 | |
| FTP age 13 → EXT age 15 | .053 | | | | | .054 | .326 | |
| *Within-time parameters* |  | | | | |  |  | |
| FTP age 17 with EXT age 17 | -.040 | | | | | .068 | .559 | |
| FTP age 15 with EXT age 15 | -.119 | | | | | .061 | .050 | |
| FTP age 13 with EXT age 13 | -.246 | | | | | .051 | <.001 | |
| **Between-person parameter:** |  | | | | |  |  | |
| FTP with EXT | -.436 | | | | | .089 | <.001 | |
| *Covariates* | | |  |  | | | |  |
| Sex→ FTP age 13 | | | .064 | .033 | | | | .049 |
| Sex→ FTP age 15 | | | .099 | .031 | | | | .002 |
| Sex→ FTP age 17 | | | .179 | .033 | | | | <.001 |
| Sex→ EXT age 13 | | | -.225 | .029 | | | | <.001 |
| Sex→ EXT age 15 | | | -.176 | .030 | | | | <.001 |
| Sex→ EXT age 17 | | | -.183 | .031 | | | | <.001 |
| Income→ FTP age 13 | | -.029 | | | .035 | | | .404 |
| Income→ FTP age 15 | | .032 | | | .034 | | | .350 |
| Income→ FTP age 17 | | -.028 | | | .037 | | | .441 |
| Income→ EXT age 13 | | -.024 | | | .034 | | | .472 |
| Income→ EXT age 15 | | -.044 | | | .035 | | | .209 |
| Income→ EXT age 17 | | -.043 | | | .035 | | | .220 |
| Parents’ education→ FTP age 13 | | .048 | | | .037 | | | .194 |
| Parents’ education→ FTP age 15 | | -.006 | | | .037 | | | .876 |
| Parents’ education→ FTP age 17 | | -.007 | | | .038 | | | .863 |
| Parents’ education→ EXT age 13 | | -.043 | | | .035 | | | .214 |
| Parents’ education→ EXT age 15 | | -.002 | | | .035 | | | .951 |
| Parents’ education→ EXT age 17 | | -.015 | | | .035 | | | .667 |
| School type→ FTP age 13 | | -.001 | | | .037 | | | .976 |
| School type→ FTP age 15 | | .095 | | | .036 | | | .009 |
| School type→ FTP age 17 | | .095 | | | .037 | | | .010 |
| School type→ EXT age 13 | | .120 | | | .030 | | | <.001 |
| School type→ EXT age 15 | | .124 | | | .027 | | | <.001 |
| School type→ EXT age 17 | | .114 | | | .029 | | | <.001 |

*Note.* FTP= future time perspective; EXT=externalising problems. The within-person parameters include autoregressive, cross-lagged, and within-time parameters.

**Table S16.** RI-CLPM standardised parameters for future time perspective and internalising problems controlling for sex, SES (household income, parents’ highest education level), and school type as covariates with time-varying effects

| **M6: INT Model controlling for sex, SES at each time point** (BIC = 9622.051) | | | | | |  |
| --- | --- | --- | --- | --- | --- | --- |
| **Within-person parameters:** | | **Estimate** | **SE** | | ***p*** |  |
| *Autoregressive parameters* | |  |  | |  |  |
| FTP age 15 → FTP age 17 | | .169 | .069 | | .014 |  |
| FTP age 13 → FTP age 15 | | .147 | .068 | | .031 |  |
| INT age 15 → INT age 17 | | .258 | .070 | | <.001 |  |
| INT age 13 → INT age 15 | | .187 | .079 | | .019 |  |
| *Cross-lagged parameters* | |  |  | |  |  |
| INT age 15 → FTP age 17 | | -.041 | .060 | | .495 |  |
| INT age 13 → FTP age 15 | | -.013 | .066 | | .844 |  |
| FTP age 15 → INT age 17 | | -.069 | .054 | | .199 |  |
| FTP age 13 → INT age 15 | | -.083 | .058 | | .150 |  |
| *Within-time parameters* | |  |  | |  |  |
| FTP age 17 with INT age 17 | | -.134 | .051 | | .009 |  |
| FTP age 15 with INT age 15 | | -.096 | .055 | | .079 |  |
| FTP age 13 with INT age 13 | | -.100 | .061 | | .101 |  |
| **Between-person parameter:** | |  |  | |  |  |
| FTP with INT | | -.178 | .093 | | .069 |  |
| *Covariates* |  | |  |  | | |
| Sex→ FTP age 13 | | .064 | .033 | | .050 | |
| Sex→ FTP age 15 | | .098 | .032 | | .002 | |
| Sex→ FTP age 17 | | .177 | .033 | | <.001 | |
| Sex→ INT age 13 | | .285 | .030 | | <.001 | |
| Sex→ INT age 15 | | .401 | .027 | | <.001 | |
| Sex→ INT age 17 | | .401 | .026 | | <.001 | |
| Income→ FTP age 13 | | -.029 | .035 | | .414 | |
| Income→ FTP age 15 | | .031 | .034 | | .370 | |
| Income→ FTP age 17 | | -.028 | .037 | | .458 | |
| Income→ INT age 13 | | .040 | .033 | | .224 | |
| Income→ INT age 15 | | .015 | .032 | | .654 | |
| Income→ INT age 17 | | .011 | .034 | | .750 | |
| Parents’ education→ FTP age 13 | | .047 | .037 | | .209 | |
| Parents’ education→ FTP age 15 | | -.005 | .037 | | .881 | |
| Parents’ education→ FTP age 17 | | -.009 | .038 | | .811 | |
| Parents’ education→ INT age 13 | | -.003 | .036 | | .929 | |
| Parents’ education→ INT age 15 | | .003 | .035 | | .938 | |
| Parents’ education→ INT age 17 | | .007 | .036 | | .848 | |
| School type→ FTP age 13 | | -.002 | .037 | | .959 | |
| School type→ FTP age 15 | | .096 | .036 | | .009 | |
| School type→ FTP age 17 | | .094 | .037 | | .012 | |
| School type→ INT age 13 | | -.039 | .035 | | .259 | |
| School type→ INT age 15 | | -.029 | .032 | | .364 | |
| School type→ INT age 17 | | -.024 | .031 | | .443 | |

*Note.* FTP= future time perspective; INT=internalising problems. The within-person parameters include autoregressive, cross-lagged, and within-time parameters.

**Section 3:**

**Model comparison, and RI-RPMs results with sex, SES (household income, Parents’ education level), and school type**

**as time-stable covariates**

**Results**

**The optimal model: RI-RPMs**

**ADHD symptoms model (Table S23, Figure 5):** Treating sex, SES and school type as time-invariant covariates, i) Autoregressive effects were all positive and significant for ADHD symptoms (age 13→15: *β* = .209, *p* < .01, [95% CI = .060, .358]; age 15→17: *β* = .299, *p* < .001, [95% CI = .165, .426]). The same held for OFTP (age 13→15: *β* = .169, *p* < .01, [95% CI = .043, .294]; age 15→17: *β* = .211, *p* < .01, [95% CI = .091, .331]). ii) Concurrent covariances between OFTP and ADHD symptoms at age 13 were negative and significant (*β* = -.136, *p* < .01, [95% CI = -.235, -.036]). iii) A reciprocal effect was only significant and negative from ADHD to OFTP at age 17 (*β* = -.310, *p* < .05, [95% CI = -.580, -.041]). iv)The random intercept factor covariance between ADHD symptoms and OFTP was significant (*r* = -.514, *p* < .001; [95% CI = -.653, -.376]). v) There were also several significant regressions of the random intercept factors on the covariates: OFTP on sex (*β* = .201, *p* < .001; [95% CI = .111, .291]), and on school type (*β* = .108, *p* < .05; [95% CI = .004, .211]); ADHD on sex (*β* = .099, *p* < .05; [95% CI = .014, .184]), and on parental education level (*β* = .101, *p* < .05; [95% CI = .002, .200]).

**Externalising problems model (Table S24, Figure 6):** Treating sex, SES and school type as time-invariant covariates, i) Autoregressive effects were all positive and significant for externalising problems (age 13→15: *β* = .271, *p* < .001, [95% CI = .137, .405]; age 15→17: *β* = .315, *p* < .01, [95% CI = .118, .511]) and OFTP (age 13→15: *β* = .135, *p* < .05, [95% CI = .005, .265]; age 15→17: *β* = .177, *p* < .01, [95% CI = .050, .305]). ii) Concurrent covariances between OFTP and externalising problems at age 13 were negative and significant (*β* = -.278, *p* < .001, [95% CI = -.369, -.188]). iii) No reciprocal effects were significant. iv)The random intercept factor covariance between externalising problems and OFTP was significant (*r* = -.315, *p* < .001; [95% CI = -.446, -.184]). v) There were also several significant regressions of the random intercept factors on the covariates: OFTP on sex (*β* = .183, *p* < .001; [95% CI = .097, .269]), and on school type (*β* = .111, *p* < .05; [95% CI = .011, .211]); externalising problems on sex (*β* = -.287, *p* < .001; [95% CI = -.369, -.205]), and on school type (*β* = .172, *p* < .001; [95% CI = .097, .246]).

**Internalising problems model (Table S25, Figure 7):** Treating sex, SES and school type as time-invariant covariates, i) Autoregressive effects were all positive and significant for internalising problems (age 13→15: *β* = .203, *p* < .01, [95% CI = .052, .354]; age 15→17: *β* = .274, *p* < .001, [95% CI = .139, .409]) and OFTP (age 13→15: *β* = .165, *p* < .05, [95% CI = .031, .299]; age 15→17: *β* = .190, *p* < .01, [95% CI = .057, .323]). ii) Concurrent covariances between OFTP and internalising problems at age 13 were not significant. iii) All reciprocal effects were not significant. iv)The random intercept factor covariance between internalising problems and OFTP was significant (*r* = -.276, *p* < .001; [95% CI = -.418, -.133]). v) There were also several significant regressions of the random intercept factors on the covariates: OFTP on sex (*β* = .202, *p* < .001; [95% CI = .114, .290]), and on school type (*β* = .109, *p* < .05; [95% CI = .008, .210]); internalising problems on sex (*β* = .540, *p* < .001; [95% CI = .463, .616]).

| **Table S17. Summary of ADHD models fit and associations (whole sample)** | | | | | | | | | |
| --- | --- | --- | --- | --- | --- | --- | --- | --- | --- |
| Model# | parameter# | LogLikelihood | BIC | CFI | TLI | RMSEA | SRMR | Sig. cross-lagged | Sig. reciprocal |
| RI-CLPM | 34 | -5134.184 | 10502.786 | 0.975 | 0.944 | 0.040 | 0.025 | TP6→ADHD7 (β=.101, *p*=.063) | NA |
| **RI-RPM** | **32** | **-5135.695** | **10492.020** | **0.975** | **0.949** | **0.038** | **0.026** | **NA** | **ADHD7→TP7 (β=-.310*, *p*=.024)** |
| RI-RCLPM reciprocal invariant | 34 | -5134.184 | 10502.786 | 0.975 | 0.944 | 0.040 | 0.025 | NO | ADHD6→TP6 (β=-.672, p=.077) ADHD7→TP7 (β=-.714, p=.054) |
| RI-RCLPM ADHD→FTP invariant | 33 | -5135.178 | 10497.880 | 0.975 | 0.945 | 0.040 | 0.027 | TP6→ADHD7 (β=.091, *p*=.099) | ADHD6→TP6 (β=-.123**, p=.003) ADHD7→TP7 (β=-.131**, p=.004) |
| RI-RCLPM ADHD→FTP | 34 | -5134.184 | 10502.786 | 0.975 | 0.944 | 0.040 | 0.025 | TP6→ADHD7 (β=.101, *p*=.063) | ADHD7→TP7 (β=-.165**, p=.001) |
| RI-RCLPM FTP→ADHD invariant | 33 | -5135.432 | 10498.388 | 0.974 | 0.944 | 0.040 | 0.027 | TP6→ADHD7 (β=.116*, *p*=.021) | TP6→ADHD6 (β=-.120, p=.005) TP7→ADHD7 (β=-.114, p=.005) |
| RI-RCLPM FTP→ADHD | 34 | -5134.184 | 10502.786 | 0.975 | 0.944 | 0.040 | 0.025 | TP6→ADHD7 (β=.128*, *p*=.011) | TP7→ADHD7 (β=-.155**, p=.001) |
| Note: RI-CLPM= random intercept cross-lagged model; RI-RPM=random intercept reciprocal panel model | | | | | | | | | |

| **Table S18. Summary of externalising problem models fit and associations (whole sample)** | | | | | | | | | |
| --- | --- | --- | --- | --- | --- | --- | --- | --- | --- |
| Model# | parameter# | LogLikelihood | BIC | CFI | TLI | RMSEA | SRMR | Sig. cross-lagged | Sig. reciprocal |
| RI-CLPM | 34 | -3492.103 | 7218.624 | 0.978 | 0.949 | 0.041 | 0.027 | TP6→EXT7 (β=.146, *p*=.050) | NA |
| **RI-RPM** | **32** | **-3495.267** | **7211.163** | **0.976** | **0.950** | **0.040** | **0.030** | **NA** | **NO** |
| RI-RCLPM reciprocal invariant | 34 | -3492.103 | 7218.624 | 0.978 | 0.949 | 0.041 | 0.027 | TP6→EXT7 (β=.128, *p*=.097) | NO |
| RI-RCLPM EXT→FTP invariant | 33 | -3492.482 | 7212.487 | 0.979 | 0.954 | 0.039 | 0.028 | TP6→EXT7 (β=.140, *p*=.053) | EXT6→TP6 (β=-.105, p=.075) EXT7→TP7 (β=-.078, p=.077) |
| RI-RCLPM EXT→FTP | 34 | -3492.103 | 7218.624 | 0.978 | 0.949 | 0.041 | 0.027 | TP6→EXT7 (β=.146, *p*=.050) | EXT6→TP6 (β=-.120, p=.054) |
| RI-RCLPM FTP→EXT invariant | 33 | -3493.419 | 7214.362 | 0.977 | 0.950 | 0.040 | 0.029 | TP6→EXT7 (β=.172*, *p*=.010) | NO |
| RI-RCLPM FTP→EXT | 34 | -3492.103 | 7218.624 | 0.978 | 0.949 | 0.041 | 0.027 | TP6→EXT7 (β=.151*, *p*=.030) | TP7→EXT7 (β=-.113, p=.054) |
| Note: RI-CLPM= random intercept cross-lagged model; RI-RPM=random intercept reciprocal panel model | | | | | | | | | |

| **Table S19. Summary of internalising problem models fit and associations (whole sample)** | | | | | | | | | |
| --- | --- | --- | --- | --- | --- | --- | --- | --- | --- |
| Model# | parameter# | LogLikelihood | BIC | CFI | TLI | RMSEA | SRMR | Sig. cross-lagged | Sig. reciprocal |
| RI-CLPM | 34 | -4664.514 | 9563.447 | 0.970 | 0.931 | 0.048 | 0.036 | NO | NA |
| **RI-RPM** | **32** | **-4664.830** | **9550.290** | **0.972** | **0.942** | **0.044** | **0.037** | **NA** | **NO** |
| RI-RCLPM reciprocal invariant | 34 | -4664.514 | 9563.447 | 0.970 | 0.931 | 0.048 | 0.036 | NO | NO |
| RI-RCLPM INT→FTP invariant | 33 | -4664.584 | 9556.692 | 0.971 | 0.937 | 0.046 | 0.036 | NO | INT6→TP6 (β=-.100*, p=.015) INT7→TP7 (β=-.110*, p=.016) |
| RI-RCLPM INT→FTP | 34 | -4664.514 | 9563.447 | 0.970 | 0.931 | 0.048 | 0.036 | NO | INT7→TP7 (β=-.119, p=.025) |
| RI-RCLPM FTP→INT invariant | 33 | -4664.717 | 9556.958 | 0.971 | 0.936 | 0.046 | 0.036 | NO | TP6→INT6 (β=-.105*, p=.017) TP7→INT7 (β=-.096*, p=.016) |
| RI-RCLPM FTP→INT | 34 | -4664.514 | 9563.447 | 0.970 | 0.931 | 0.048 | 0.036 | NO | TP7→INT7 (β=-.113*, p=.025) |
| Note: RI-CLPM= random intercept cross-lagged model; RI-RPM=random intercept reciprocal panel model | | | | | | | | | |

**Table S20.** Correlations controlling for sex, SES (household income, parents’ highest education level), and school type among future time perspective and ADHD at the between- and within-person levels

|  | 1 | 2 | 6 | 7 | 8 | 9 | 10 | 11 |
| --- | --- | --- | --- | --- | --- | --- | --- | --- |
| **Between-person correlations** |  |  |  |  |  |  |  |  |
| 1. RI_FTP | _ |  |  |  |  |  |  |  |
| 2. RI_ADHD | -.483*** | _ |  |  |  |  |  |  |
| 3. Sex | .196*** | .100* | _ | _ | _ | _ | _ | _ |
| 4. Household income | -.045 | .062 | _ | _ | _ | _ | _ | _ |
| 5. Parents’ education level | -.032 | .125** | _ | _ | _ | _ | _ | _ |
| 6. School type | .096* | -.100* | _ | _ | _ | _ | _ | _ |
| **Within-person correlations** |  |  |  |  |  |  |  |  |
| 6. Age 13 FTP | _ | _ | _ |  |  |  |  |  |
| 7. Age 15 FTP | _ | _ | .171** | _ |  |  |  |  |
| 8. Age 17 FTP | _ | _ | .038 | .214*** | _ |  |  |  |
| 9. Age 13 ADHD | _ | _ | -.136** | -.034 | -.026* | _ |  |  |
| 10. Age 15 ADHD | _ | _ | .039 | -.124** | -.114** | .211** | _ |  |
| 11. Age 17 ADHD | _ | _ | -.007 | -.010 | -.203*** | .060 | .284*** | _ |

*Note.* RI*=*Random-Intercept; FTP= future time perspective; **p*<.05; ***p*<.01; ****p*<.001.

**Table S21.** Correlations controlling for sex, SES (household income, parents’ highest education level), and school type among future time perspective and externalising problems at the between- and within-person levels

|  | 1 | 2 | 6 | 7 | 8 | 9 | 10 | 11 |
| --- | --- | --- | --- | --- | --- | --- | --- | --- |
| **Between-person correlations** |  |  |  |  |  |  |  |  |
| 1. RI_FTP | _ |  |  |  |  |  |  |  |
| 2. RI_EXT | -.318*** | _ |  |  |  |  |  |  |
| 3. Sex | .178*** | -.294*** | _ | _ | _ | _ | _ | _ |
| 4. Household income | -.044 | -.120** | _ | _ | _ | _ | _ | _ |
| 5. Parents’ education level | -.033 | -.120** | _ | _ | _ | _ | _ | _ |
| 6. School type | .099* | .214*** | _ | _ | _ | _ | _ | _ |
| **Within-person correlations** |  |  |  |  |  |  |  |  |
| 6. Age 13 FTP | _ | _ | _ |  |  |  |  |  |
| 7. Age 15 FTP | _ | _ | .148* | _ |  |  |  |  |
| 8. Age 17 FTP | _ | _ | .029 | .184** | _ |  |  |  |
| 9. Age 13 EXT | _ | _ | -.278*** | -.080 | -.024 | _ |  |  |
| 10. Age 15 EXT | _ | _ | -.082* | -.207** | -.071 | .275*** | _ |  |
| 11. Age 17 EXT | _ | _ | -.026 | -.064 | -.115 | .086 | .314** | _ |

*Note.* RI*=*Random-Intercept; FTP= future time perspective; EXT=externalising problems; **p*<.05; ***p*<.01; ****p*<.001.

**Table S22.** Correlations controlling for sex, SES (household income, parents’ highest education level), and school type among future time perspective and internalising problems at the between- and within-person levels

|  | 1 | 2 | 6 | 7 | 8 | 9 | 10 | 11 |
| --- | --- | --- | --- | --- | --- | --- | --- | --- |
| **Between-person correlations** |  |  |  |  |  |  |  |  |
| 1. RI_FTP | _ |  |  |  |  |  |  |  |
| 2. RI_INT | -.124 | _ |  |  |  |  |  |  |
| 3. Sex | .198*** | .542*** | _ | _ | _ | _ | _ | _ |
| 4. Household income | -.045 | .043 | _ | _ | _ | _ | _ | _ |
| 5. Parents’ education level | -.035 | .027 | _ | _ | _ | _ | _ | _ |
| 6. School type | .098* | -.079* | _ | _ | _ | _ | _ | _ |
| **Within-person correlations** |  |  |  |  |  |  |  |  |
| 6. Age 13 FTP | _ | _ | _ |  |  |  |  |  |
| 7. Age 15 FTP | _ | _ | .155* | _ |  |  |  |  |
| 8. Age 17 FTP | _ | _ | .029 | .188** | _ |  |  |  |
| 9. Age 13 INT | _ | _ | -.046 | .032 | .009 | _ |  |  |
| 10. Age 15 INT | _ | _ | -.049 | -.054 | .004 | .195* | _ |  |
| 11. Age 17 INT | _ | _ | -.018 | -.043 | -.101* | .052 | .273*** | _ |

*Note.* RI*=*Random-Intercept; FTP= future time perspective; INT=internalising problems; **p*<.05; ***p*<.01; ****p*<.001.

**Table S23.** RI-RPM standardised parameters for future time perspective and ADHD controlling for sex, SES (household income, parents’ highest education level), and school type as covariates with time-stable effects

| **M7: ADHD RI-RPM Model controlling for sex, SES, school type at random intercept** (BIC = 10492.020) | | | | | | |
| --- | --- | --- | --- | --- | --- | --- |
| **Within-person parameters:** | | **Estimate [95%CI]** | **SE** | | | ***p*** |
| *Autoregressive parameters* | |  |  | | |  |
| FTP age 15 → FTP age 17 | | .211 [.091, .331] | .061 | | | .001 |
| FTP age 13 → FTP age 15 | | .169 [.043, .294] | .064 | | | .008 |
| ADHD age 15 → ADHD age 17 | | .299 [.168, .429] | .067 | | | <.001 |
| ADHD age 13 → ADHD age 15 | | .209 [.060, .358] | .076 | | | .006 |
| *Reciprocal parameters* |  | | |  |  | |
| ADHD age 17 → FTP age 17 | | -.310 [-.580, -.041] | .138 | | | .024 |
| ADHD age 15 → FTP age 15 | | -.055 [-.390, .280] | .171 | | | .749 |
| FTP age 17 → ADHD age 17 | | .123 [-.166, .412] | .147 | | | .403 |
| FTP age 15 → ADHD age 15 | | -.065 [-.387, .258] | .165 | | | .695 |
| *Within-time parameters* | |  |  | | |  |
| FTP age 13 with ADHD age 13 | | -.136 [-.235, -.036] | .051 | | | .007 |
| **Between-person parameter:** | |  |  | | |  |
| FTP with ADHD | | -.514 [-.653, -.376] | .071 | | | <.001 |
| *Covariates* | |  |  | | |  |
| Sex→ FTP | | .201 [.111, .291] | .046 | | | < .001 |
| Income→ FTP | | -.018 [-.112, .076] | .048 | | | .711 |
| Parents’ education→ FTP | | .022 [-.078, .123] | .051 | | | .665 |
| School type→ FTP | | .108 [.004, .211] | .053 | | | .042 |
| Sex→ ADHD | | .099 [.014, .184] | .043 | | | .023 |
| Income→ ADHD | | .008 [-.083, .100] | .047 | | | .861 |
| Parents’ education→ ADHD | | .101 [.002, .200] | .050 | | | .045 |
| School type→ ADHD | | -.050 [-.146, .047] | .049 | | | .312 |

*Note.* FTP= future time perspective. The within-person parameters include autoregressive, cross-lagged, and within-time parameters.

**Table S24.** RI-RPM standardised parameters for future time perspective and externalising problems controlling for sex, SES (household income, parents’ highest education level), and school type as covariates with time-stable effects

| **M8: EXT RI-RPM Model controlling for sex, SES, and School type at random intercept** (BIC = 7211.163) | | | |
| --- | --- | --- | --- |
| **Within-person parameters:** | **Estimate [95%CI]** | **SE** | ***p*** |
| *Autoregressive parameters* |  |  |  |
| FTP age 15 → FTP age 17 | .177 [.050, .305] | .065 | .006 |
| FTP age 13 → FTP age 15 | .135 [.005, .265] | .066 | .042 |
| EXT age 15 → EXT age 17 | .315 [.118, .511] | .100 | .002 |
| EXT age 13 → EXT age 15 | .271 [.137, .405] | .068 | <.001 |
| *Reciprocal parameters* |  |  |  |
| EXT age 17 → FTP age 17 | -.108 [-.392, .176] | .145 | .455 |
| EXT age 15 → FTP age 15 | -.154 [-.470, .162] | .161 | .340 |
| FTP age 17 → EXT age 17 | .004 [-.256, .265] | .133 | .973 |
| FTP age 15 → EXT age 15 | -.045 [-.360, 270] | .161 | .782 |
| *Within-time parameters* |  |  |  |
| FTP age 13 with EXT age 13 | -.278 [-.369, -.188] | .046 | <.001 |
| **Between-person parameter:** |  |  |  |
| FTP with EXT | -.315 [-.446, -.184] | .067 | <.001 |
| *Covariates* |  |  |  |
| Sex→ FTP | .183 [.097, .269] | .044 | < .001 |
| Income→ FTP | -.015 [-.106, .075] | .046 | .741 |
| Parents’ education→ FTP | .021 [-.076, .118] | .049 | .673 |
| School type→ FTP | .111 [.011, .211] | .051 | .029 |
| Sex→ EXT | -.287 [-.369, -.205] | .042 | < .001 |
| Income→ EXT | -.057 [-.139, .024] | .041 | .168 |
| Parents’ education→ EXT | -.027 [-.112, .058] | .043 | .531 |
| School type→ EXT | .172 [.097, .246] | .038 | < .001 |

*Note.* FTP= future time perspective; EXT=externalising problems. The within-person parameters include autoregressive, cross-lagged, and within-time parameters.

**Table S25.** RI-RPM standardised parameters for future time perspective and internalising problems controlling for sex, SES (household income, parents’ highest education level), and school type as covariates with time-stable effects

| **M9: INT RI-RPM Model controlling for sex, SES, School type at random intercept** (BIC = 9550.290) | | | |
| --- | --- | --- | --- |
| **Within-person parameters:** | **Estimate [95% CI]** | **SE** | ***p*** |
| *Autoregressive parameters* |  |  |  |
| FTP age 15 → FTP age 17 | .190 [.057, .323] | .068 | .005 |
| FTP age 13 → FTP age 15 | .165 [.031, .299] | .069 | .016 |
| INT age 15 → INT age 17 | .274 [.139, .409] | .069 | <.001 |
| INT age 13 → INT age 15 | .203 [.052, .354] | .077 | .008 |
| *Reciprocal parameters* |  |  |  |
| INT age 17 → FTP age 17 | .052 [-.233, .336] | .145 | .723 |
| INT age 15 → FTP age 15 | .204 [-.254, .663] | .234 | .382 |
| FTP age 17 → INT age 17 | -.149 [-.411, .112] | .133 | .263 |
| FTP age 15 → INT age 15 | -.254 [-.695, .187] | .225 | .259 |
| *Within-time parameters* |  |  |  |
| FTP age 13 with INT age 13 | -.046 [-.141, .048] | .048 | .337 |
| **Between-person parameter:** |  |  |  |
| FTP with INT | -.276 [-.418, -.133] | .073 | <.001 |
| *Covariates* |  |  |  |
| Sex→ FTP | .202 [.114, .290] | .045 | < .001 |
| Income→ FTP | -.016 [-.108, .075] | .047 | .726 |
| Parents’ education→ FTP | .019 [-.079, .118] | .050 | .703 |
| School type→ FTP | .109 [.008, .210] | .052 | .035 |
| Sex→ INT | .540 [.463, .616] | .039 | < .001 |
| Income→ INT | .033 [-.044, .109] | .039 | .401 |
| Parents’ education→ INT | -.002 [-.086, .082] | .043 | .956 |
| School type→ INT | -.047 [-.125, .032] | .040 | .244 |

*Note.* FTP= future time perspective; INT=internalising problems. The within-person parameters include autoregressive, cross-lagged, and within-time parameters.
